# Supplementary material for: Natural barriers: waterfall transit by small flying animals
Source: R Soc Open Sci. 2020 Aug 19;7(8):201185. doi: 10.1098/rsos.201185 (PMC7481727; doi:10.1098/rsos.201185)
Supplement: Supplementary information [file rsos201185supp1.pdf]

SUPPLEMENTARY MATERIALS

Natural Barriers: Waterfall Transit by Small Flying Animals

Victor M. Ortega-Jimenez[1], Eva C. Herbst[2], Michelle S. Leung[3], Robert Dudley[1,4]

[1]Department of Integrative Biology, University of California, Berkeley, CA 94720 USA
[2]Palaeontological Institute and Museum, University of Zurich, Switzerland
[3]University of California, San Francisco School of Medicine, San Francisco, CA 94143 USA
[4]Smithsonian Tropical Research Institute, Balboa, Republic of Panama
[*]Author for correspondence (ornithopterus@gmail.com)

Table S1. Morphometric data from four Anna's Hummingbirds, a bottle fly (*Lucilia sp*), a house fly (*Musca sp*), crane fly (*Tipula sp*) , and a fruit fly (*Drosophila melanogaster*). Fruit fly mass was estimated as an eighth of the total mass of 8 individuals. Hummingbirds data represent average value ± one standard deviation.

Sp	Body mass (g)	Wing length (cm)	One-wing area (cm^2)
Anna's Hummingbird	5.0±0.4 g	5.3±0.2	7.4±0.4
Bottle Fly	0.06	1.1	0.30
House Fly	0.02	0.8	0.17
Crane Fly	0.31	1.9	0.67
Fruit Fly	0.001	0.2	0.01

Table S2. Pairwise comparisons of kinematic variables of hummingbirds (n=4) among control flights and before, during and after crossing the waterfall.

**Pitch**			Estimate	SE	Z value	Pr(>|z|)	
before	-	after	-29.17	4.62	-6.32	<1e-04	***
control	-	after	-26.84	4.62	-5.81	<1e-04	***
during	-	after	-21.16	4.62	-4.59	<1e-04	***
control	-	before	2.33	4.62	0.51	0.96	
during	-	before	8.01	4.62	1.73	0.31	
during	-	control	5.67	4.62	1.23	0.61	
**Roll**			Estimate	SE	Z value	Pr(>|z|)	
before	-	after	18.02	5.52	3.27	0.01	**
control	-	after	10.35	5.52	1.87	0.24	
during	-	after	20.25	5.52	3.67	0.001	**
control	-	before	-7.68	5.52	-1.39	0.5	
during	-	before	2.23	5.52	0.4	0.98	
during	-	control	9.9	5.52	1.79	0.28	
**Yaw**			Estimate	SE	Z value	Pr(>|z|)	
before	-	after	-42.39	10.18	-4.16	<0.001	***
control	-	after	-45.5	10.18	-4.47	<0.001	***
during	-	after	-28.36	10.18	-2.79	0.03	*
control	-	before	-3.11	10.18	-0.31	0.99	
during	-	before	14.04	10.18	1.38	0.51	
during	-	control	17.14	10.18	1.68	0.33	
**Stroke amplitude**			Estimate	SE	Z value	Pr(>|z|)	
before	-	after	-3.32	8.52	-0.39	0.98	
control	-	after	21.68	8.52	2.54	0.05	
during	-	after	-4.36	8.52	-0.51	0.96	
control	-	before	25	8.52	2.93	0.02	*
during	-	before	-1.05	8.52	-0.12	1	
during	-	control	-26.04	8.52	-3.06	0.01	*
**Tail pitch**			Estimate	SE	Z value	Pr(>|z|)	
before	-	after	-32.96	6.85	-4.81	<1e-04	***
control	-	after	-33.03	6.85	-4.83	<1e-04	***
during	-	after	-39.75	6.85	-5.81	<1e-04	***
control	-	before	-0.07	6.85	-0.01	1	
during	-	before	-6.79	6.85	-0.99	0.75	
during	-	control	-6.72	6.85	-0.98	0.76	

**Tail spread**			Estimate	SE	Z value	Pr(>|z|)	
before	-	after	-26.58	7.32	-3.63	0.002	**
control	-	after	-44.71	7.32	-6.11	<0.001	***
during	-	after	-8.62	7.32	-1.18	0.64	
control	-	before	-18.13	7.32	-2.48	0.06	
during	-	before	17.96	7.32	2.45	0.07	
during	-	control	36.09	7.32	4.93	<0.001	***
**Speed**			Estimate	SE	Z value	Pr(>|z|)	
before	-	after	0.3	0.11	2.77	0.03	*
control	-	after	0.46	0.11	4.27	<0.001	***
during	-	after	0.19	0.11	1.81	0.27	
control	-	before	0.16	0.11	1.5	0.44	
during	-	before	-0.1	0.11	-0.96	0.77	
during	-	control	-0.26	0.11	-2.46	0.07	
**Acceleration**			P-Value (Friedman Post Hoc)				
before	-	after	0.99				
control	-	after	0.07				
during	-	after	0.69				
control	-	before	0.03	*			
during	-	before	0.52				
during	-	control	0.52				

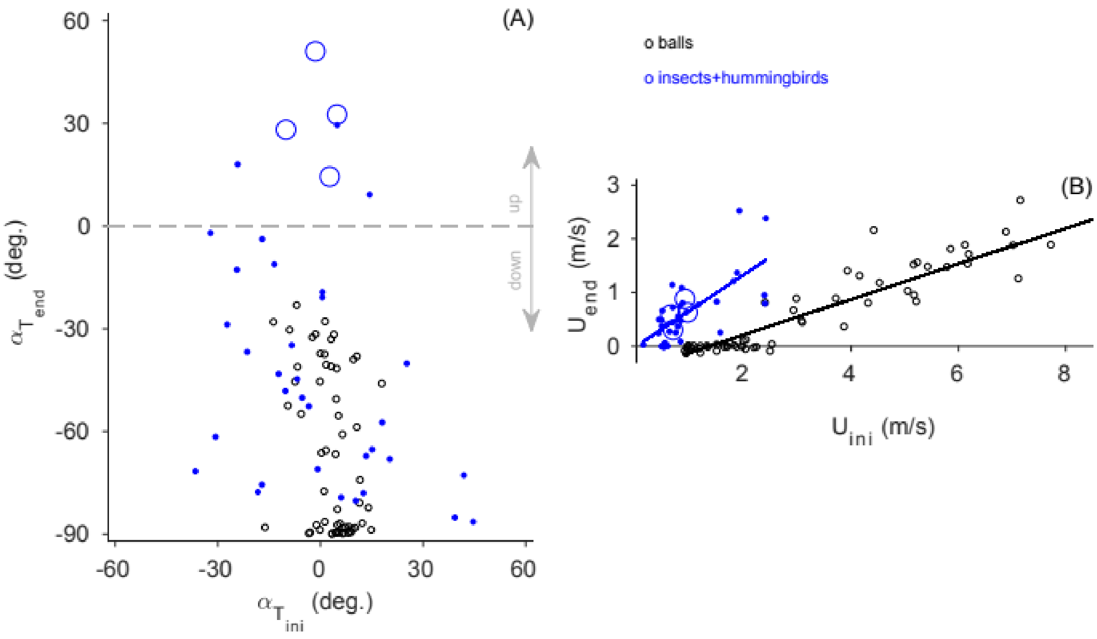

Figure S1. (A) initial trajectory angle α_{Tini} versus final trajectory angle α_{Tend} for 33 insects (blue dots) and 4 hummingbirds (blue open circles), and for plastic balls (n=59, black circles) interacting with the waterfall. (B) Initial speed (U_{ini}) versus final speed (U_{end}) at the end of the trial for 33 insects and 4 hummingbirds (r^2=0.5, F=33.6, p<0.001, blue line), and for 59 plastic balls (r^2=0.85 F=318, p<0.001, dark line) interacting with the waterfall. Initial trajectory angle to to waterfall sheet was calculated using the ten positional values immediately preceding waterfall transit, and for the ten values at the end of the recorded positional data.

Supplementary Videos

VideoS1.mp4 [(00:12 s) Hummingbird flying asymmetrically through an artificial waterfall;(00:40 s) Hummingbird flying symmetrically through an artificial waterfall, with attached water droplets post-transit; (01:01 s) Insects, styrofoam balls, and a plastic plate interacting with the waterfall]

VideoS2.mp4 [(00:1 s) Foam balls of three sizes (1 to 8 cm diameter) launched against a waterfall; (00:10 s) Foam ball (4 cm diameter) launched at high speed and demonstrating marked splashing]

Raw Data

Raw_data_OrtegaJimenezetal.xls [Data sets and raw digitization data. (sheet 1) Hummingbirds' kinematics. (Sheet 2) insects' kinematics. (sheet 3) balls' kinematics. (sheet 4-11) 3D digitized points in Cartesian system XYZ in cm of four male hummingbirds flying in still air (control) and crossing through a waterfall. Point 1 (beak), point 2 (eye), point3 (tail base), point 4 (tail tip at the middle), point5 (right wingtip), point 6 (right shoulder), point 7 (left shoulder), point8 (left wingtip), point 9 (right tailtip). (sheet 12-44) 3D digitized points in Cartesian system XYZ in cm of 33 insects (7 bottleflies 24 house flies, one cranefly and one fruit fly crossing through a waterfall. Point 1 (head) and point 2 (abdomen).(sheet 45-46) 2D trajectories in Cartesian system XY in cm of 59 plastic balls shot against a waterfall. Digitalization software is described in: Hedrick, T. L. (2008). Software techniques for two- and three-dimensional kinematic measurements of biological and biomimetic systems. Bioinspir. Biomim. 3, 034001].
